# Endoleak type IIIb not always recognized

**DOI:** 10.1016/j.jvscit.2026.102350

**Published:** 2026-06-05

**Authors:** Mahmoud Tayeh, Mohammad Sharkawy, Adel Aswad, Payman Majd

**Affiliations:** a Division of Vascular and Endovascular Surgery, Evangelic Hospital Bergisch Gladbach, Bergisch Gladbach, Germany; b Division of Thorcic and Vascular Surgery, Al-Qassim Hospital, Buraydah, UAE; c Division of Vascular Surgery, University Hospital Cairo, Cairo, Egypt

**Keywords:** Abdominal aortic aneurysm, Type IIIb endoleak, EVAR, Open repair, Graft defect

## Abstract

Endovascular aneurysm repair (EVAR) is a standard treatment for abdominal aortic aneurysms but is associated with late graft-related complications. Type IIIb endoleaks caused by graft fabric failure are rare and potentially fatal. We report two cases of late type IIIb endoleaks occurring 60 and 48 months after endovascular aneurysm repair using Endurant (Medtronic, Inc.) and GORE EXCLUDER (W. L. Gore & Associates, Inc.) stent grafts, respectively. Diagnosis was challenging because of limited imaging sensitivity. Both cases highlight the persistent risk of late graft failure and underscore the continued role of open surgical repair as a definitive treatment option.

Endovascular aneurysm repair (EVAR) has emerged as a preferred treatment modality for abdominal aortic aneurysms (AAA) due to its significantly lower perioperative mortality compared with open repair. Since the first EVAR was performed in 1991, it has become an established therapeutic option.^1^

Fifteen years later, EVAR was used more frequently than open repair, largely due to its reduced operative mortality. However, higher rates of graft-related complications, functional impairment, and reinterventions have been observed following EVAR.^2^

Long-term complications, particularly graft-related failures such as endoleaks, remain a major concern. Type IIIb endoleaks, caused by defects in the graft material, are especially dangerous because they result in direct high-pressure arterial blood flow into the aneurysm sac, significantly increasing the risk of rupture.^3^

Management of ruptured AAAs after EVAR is challenging, as no standardized treatment guidelines exist for this specific scenario. Most available evidence is derived from small case series and retrospective studies, limiting the ability to establish definitive treatment strategies.^4^, ^5^, ^6^

## Case 1

A 75-year-old man was admitted to our emergency department with recurrent, nonspecific abdominal pain. His medical history was significant for hypertension, coronary artery disease, previous stroke, nephrectomy, chronic pyelonephritis, and previous EVAR for a 56-mm AAA. The EVAR procedure had been performed 60 months earlier at another vascular center using an Endurant stent graft (Medtronic Cardiovascular). Notably, the patient had been lost to follow-up, and no surveillance imaging had been performed since the intervention.

**Fig 1:**
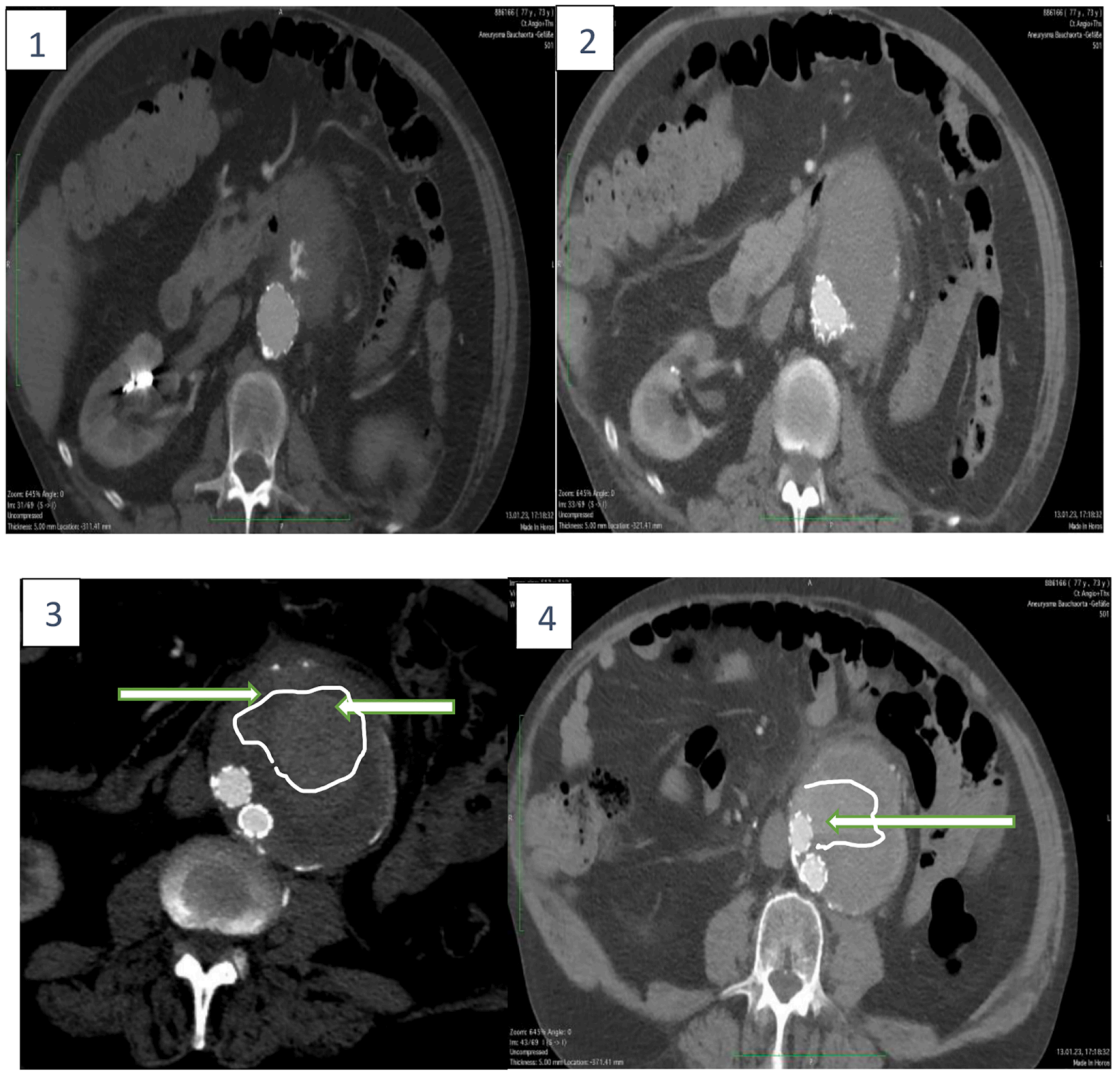
Computed tomography (CT) scan at admission: patient 1.

At presentation, owing to nonspecific abdominal pain and known pyelonephritis, a computed tomography (CT) scan was obtained after an ultrasound study demonstrated an aneurysm diameter of 80 mm. CT imaging revealed aneurysm expansion to 85 mm with suspicion of a type II endoleak and mosaic signs within the aneurysm sac (white arrow, Fig 1).

**Fig 2:**
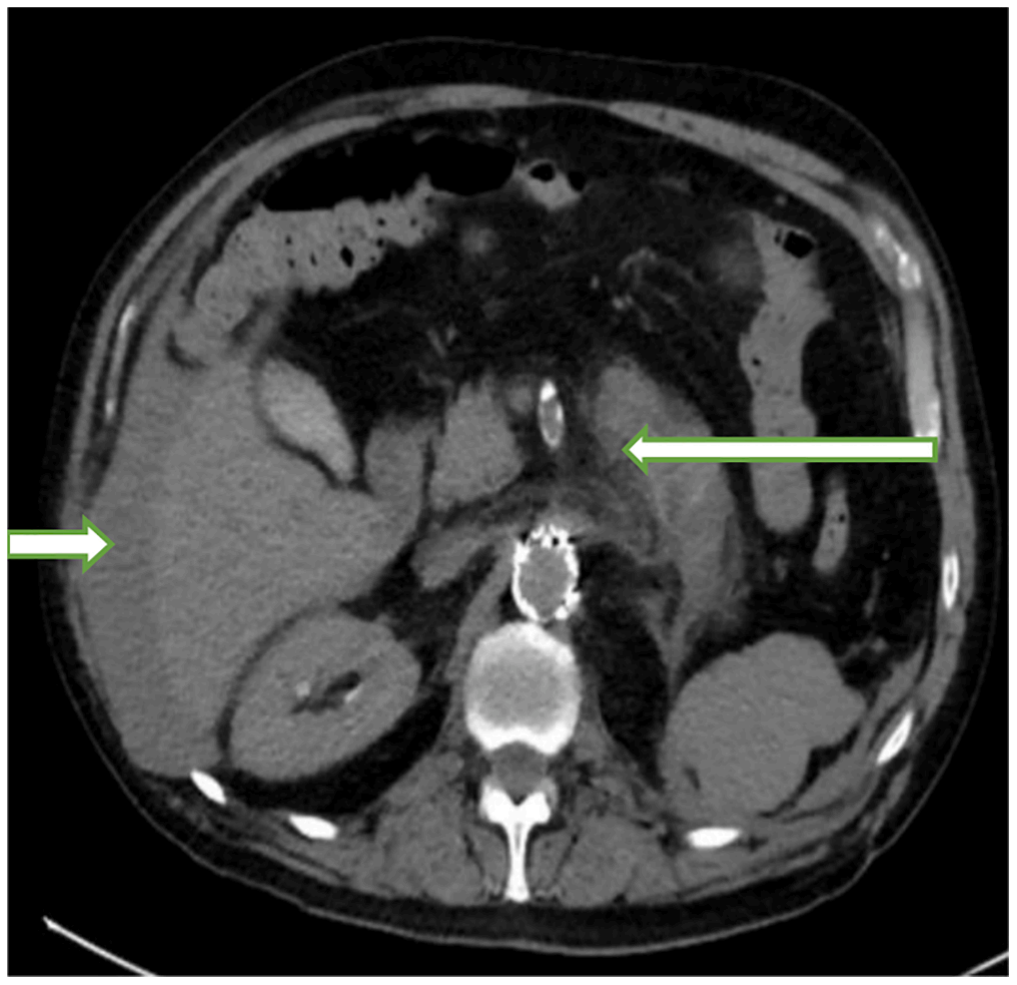
Computed tomography (CT) scan 8 hours later: patient 1.

On the evening of admission, the patient collapsed. Emergency ultrasound study revealed free intra-abdominal fluid, prompting urgent contrast-enhanced CT imaging, which confirmed retroperitoneal and perihepatic hematoma consistent with aneurysm rupture (Fig 2). The patient was immediately transferred to the operating room.

**Fig 3:**
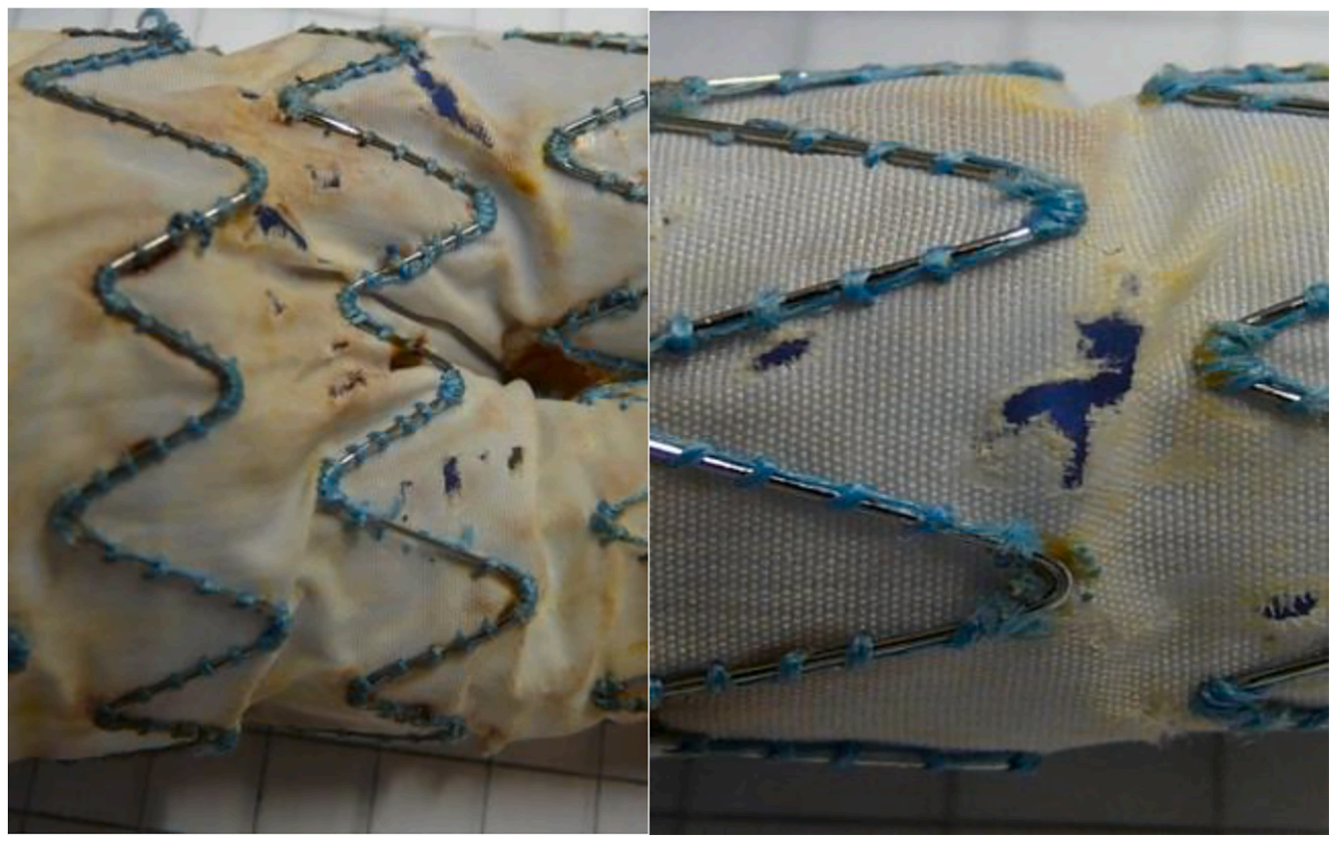
Defects at the endoprothesis: patient 1.

Given the aneurysm enlargement to 85 mm and confirmed rupture, because of the rupture, the indication for treatment was established, so an angiography was initially performed, which revealed bleeding from right iliac limb at Flow divider. An iliac limb was then implanted (PLC 181200 Gore AAA endoprosthesis). However, in the follow-up angiography, bleeding at more spots was still observed, so the indication for an open surgery was established. After infrarenal aortic clamping, the aneurysm sac was opened, revealing defects in the main body of the stent graft and at the flow divider (Fig 3). The endograft was explanted, leaving the suprarenal fixation in situ, and an aortobiiliac prosthesis was successfully implanted.

The postoperative course was prolonged, requiring a second-look operation following abdominal packing, sigmoid resection with protective ileostomy, and placement of a Demers catheter for hemodialysis.

After approximately 2 months of hospitalization, the patient was discharged. At 6-month follow-up, the ileostomy had been reversed; however, the patient remained dialysis dependent.

## Case 2

A 62-year-old man underwent EVAR for an expanding 69-mm AAA 48 months earlier using a GORE EXCLUDER AAA (W. L. Gore & Associates, Inc.) endoprosthesis. The patient had initially declined open repair. His medical history included hypertension, obesity, tobacco use, and antiplatelet therapy with aspirin 100 mg daily.

**Fig 4:**
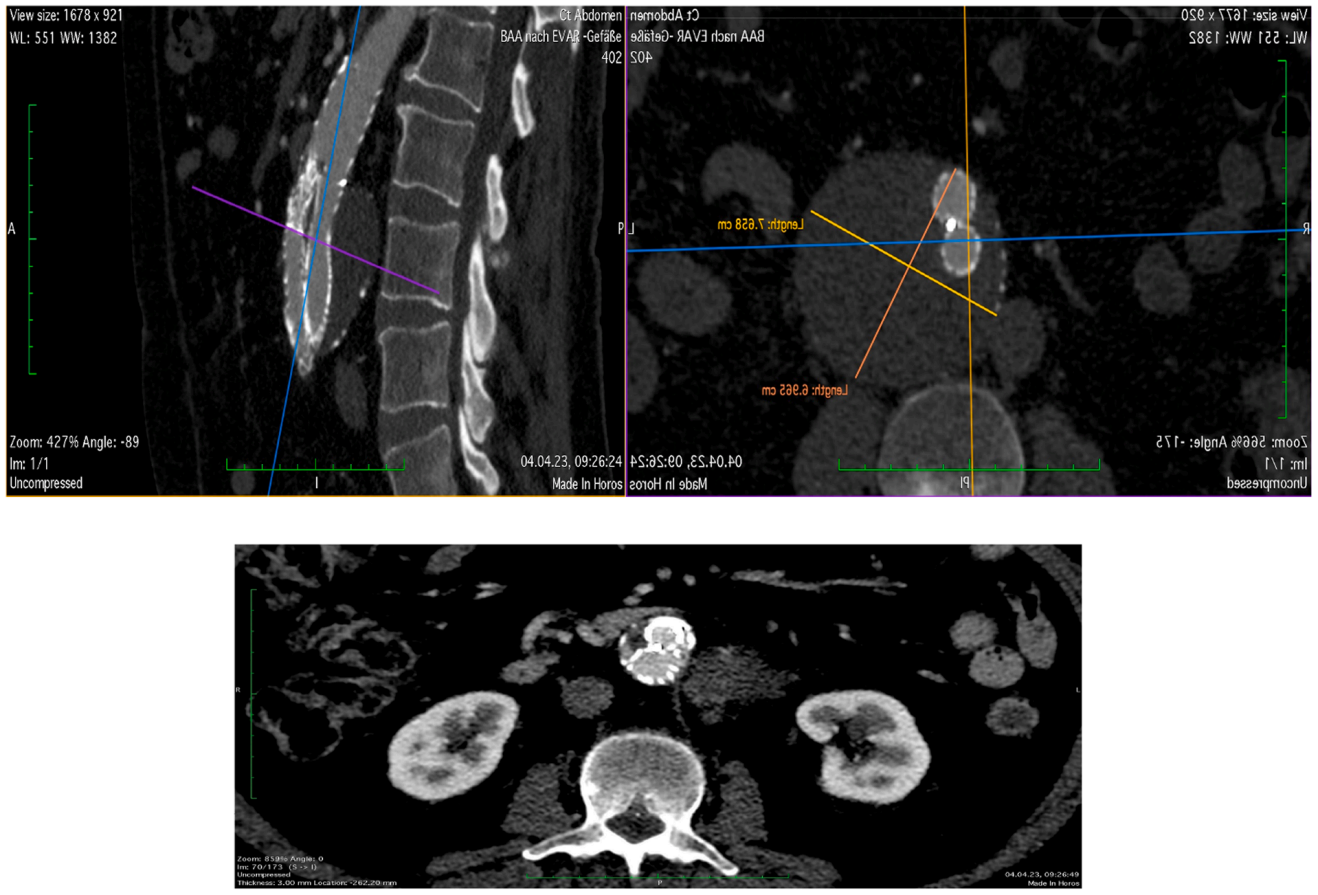
Computed tomography (CT) scan of patient 2.

**Fig 5:**
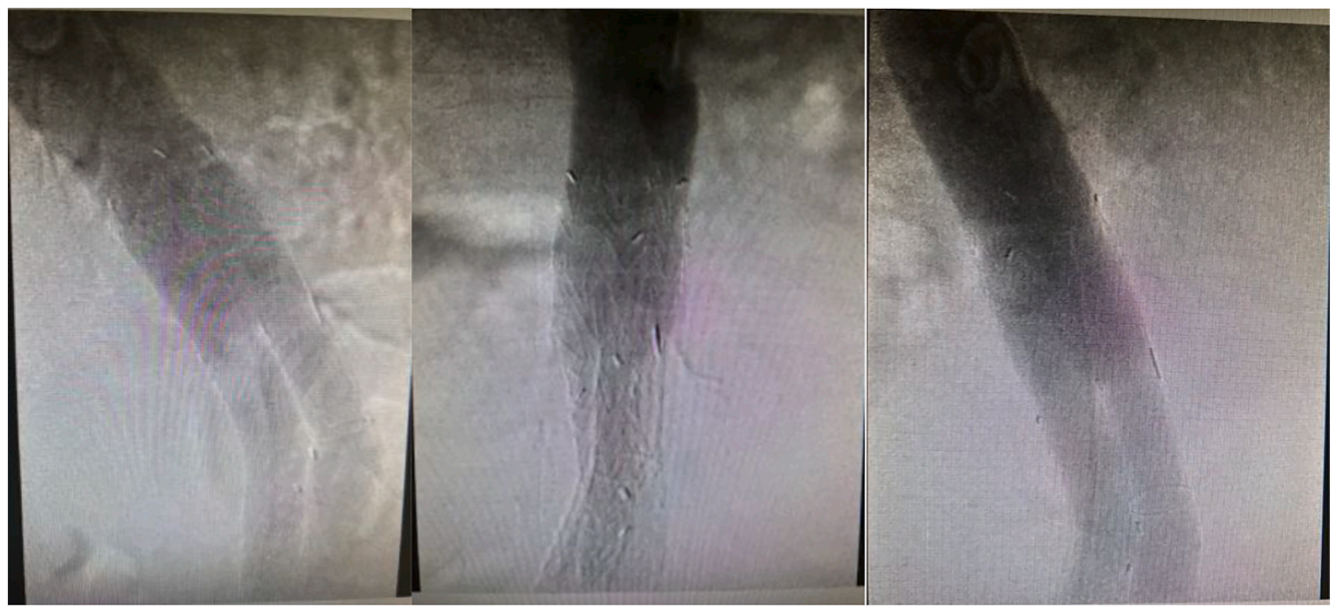
Angiogram of patient 2 showing no endoleak.

Routine follow-up at 6, 12, 24, and 36 months demonstrated progressive aneurysm sac shrinkage on ultrasound study. At 48 months, ultrasound study detected aneurysm sac enlargement to 80 mm, raising suspicion of a type II endoleak after performing a CT scan (Fig 4). Subsequent CT angiography did not provide definitive findings. Diagnostic angiography was therefore performed to exclude type I or III endoleaks, but no clear endoleak was identified (Fig 5).

**Fig 6:**
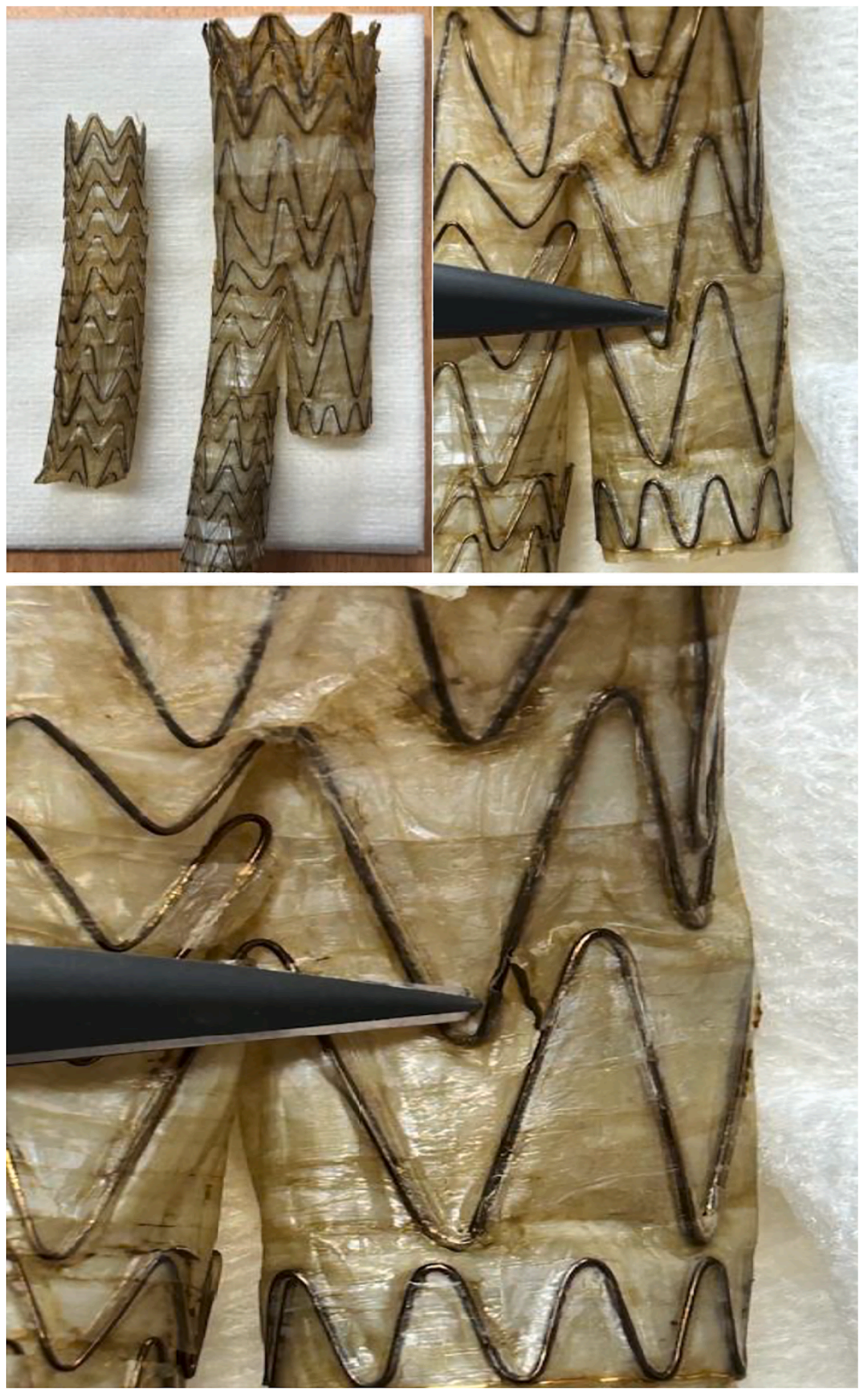
Defects at the endoprothesis: patient 2.

Given the unclear imaging results with no visible endoleak at the CT scan or angiography, the patient's relatively young age, and good general condition, elective open surgical conversion was performed after informed consent. Following explantation of the endograft, inspection revealed a microperforation in the main body of the graft (Fig 6). An aortobi-iliac prosthesis was implanted. The postoperative course was uneventful, and the patient was discharged after 14 days.

## Discussion

EVAR has become the standard treatment for AAA because of reduced perioperative morbidity and mortality compared with open repair. Nevertheless, long-term complications such as endoleaks—particularly type IIIb—represent a serious and potentially fatal challenge. Type IIIb endoleaks result from graft material failure, including fabric tears or holes, allowing pressurized arterial blood to enter the aneurysm sac and significantly increasing rupture risk.

A graft defect is classified as a type IIIb endoleak and is defined by fabric disruption or midgraft holes.^4^^,^^7^ These defects are often attributed to material fatigue rather than erosion. Similar to type I endoleaks, type III endoleaks are high-pressure lesions and therefore require urgent treatment to prevent rupture.^7^

Management options include relining the graft with additional endovascular components, conversion to an aortouni-iliac system with femorofemoral bypass, or open surgical conversion when endovascular options are not feasible or fail.^4^^,^^7^

Rupture after EVAR is a life-threatening emergency requiring immediate intervention. Evidence from small series suggests that open repair is often chosen in this context, although operative risk is high due to patient comorbidities and technical challenges related to the existing endograft.^4^^,^^6^ Endovascular repair of secondarily ruptured AAAs after EVAR remains rare and poorly documented.^5^^,^^8^

When a type IIIb endoleak is identified, management typically involves empiric endovascular relining of the graft.^9^ If endovascular repair is unsuccessful or infeasible, open surgical conversion is required. A systematic review by Lowe et al^10^ reported that 69% of endoleaks were treated endovascularly; however, in cases of secondary rupture after EVAR, open repair is often favored due to the urgency and technical complexity of the situation.

In a multicenter study by Geert et al^9^ involving 965 patients with EVAR, 88% of type III endoleaks were managed endovascularly, whereas 12% required open conversion.

Despite high technical success, recurrence occurred in 25% of patients after endovascular treatment. Open conversion was reserved for severe complications, including aneurysm rupture and aortoduodenal fistula.^9^^,^^11^

Endovascular management of secondary ruptures after EVAR may be limited by device availability, anatomical constraints, and operator experience.^4^ Therefore, vascular centers should maintain a basic stock of stent grafts, iliac extensions, and proximal cuffs, and ensure expertise in advanced techniques such as aortic occlusion balloon deployment and conversion to aortouni-iliac systems.^4^

Type III endoleaks can occur at any time after EVAR and may remain asymptomatic, underscoring the importance of lifelong imaging surveillance.^9^ Although long-term survival after EVAR and open repair appears comparable, EVAR is associated with a higher risk of reintervention and secondary rupture, which remains a critical limitation of this approach.^2^^,^^12^

## Conclusions

No endovascular device is entirely free from late device-related complications, which can be potentially life-threatening and difficult to detect. A key indicator of concern is the presence of a high-density area in imaging studies, along with an enlargement of the aneurysm sac.^7^

Even in the absence of a visible endoleak, these findings should prompt consideration of reintervention. Maintaining vigilance is essential, as early identification and proactive management of such complications can prevent further deterioration and improve patient outcomes.^7^

## Funding

None.

## Informed consent

Written informed consent was obtained from the patients for publication of the case details and accompanying images.

## Disclosures

None.
